# Epigenetic Regulation Mediated by Sphingolipids in Cancer

**DOI:** 10.3390/ijms24065294

**Published:** 2023-03-10

**Authors:** Nicolò Bozzini, Sofia Avnet, Nicola Baldini, Margherita Cortini

**Affiliations:** 1 Biomedical Science and Technologies and Nanobiotecnologiy Lab., IRCCS Istituto Ortopedico Rizzoli, 40136 Bologna, Italy; 2 Department of Biomedical and Neuromotor Sciences, University of Bologna, 40126 Bologna, Italy

**Keywords:** epigenetics, cancer, sphingolipids, tumour microenvironment, hypoxia, acidosis, bone cancer

## Abstract

Epigenetic changes are heritable modifications that do not directly affect the DNA sequence. In cancer cells, the maintenance of a stable epigenetic profile can be crucial to support survival and proliferation, and said profile can differ significantly from that of healthy cells. The epigenetic profile of a cancer cell can be modulated by several factors, including metabolites. Recently, sphingolipids have emerged as novel modulators of epigenetic changes. Ceramide and sphingosine 1-phosphate have become well known in cancer due to activating anti-tumour and pro-tumour signalling pathways, respectively, and they have recently been shown to also induce several epigenetic modifications connected to cancer growth. Additionally, acellular factors in the tumour microenvironment, such as hypoxia and acidosis, are now recognised as crucial in promoting aggressiveness through several mechanisms, including epigenetic modifications. Here, we review the existing literature on sphingolipids, cancer, and epigenetic changes, with a focus on the interaction between these elements and components of the chemical tumour microenvironment.

## 1. Introduction

Tumour development and growth can depend on many factors that often differ from cancer to cancer. Despite this, it is widely recognised that, in order to evade the organism’s safety checks, primarily apoptosis, all cancer cells must acquire a number of genomic alterations. However, said alterations are not solely dependent on direct genomic mutations, since changes in expression might also derive from an altered epigenetic profile, and lead to similar consequences. Epigenetic changes are heritable modifications that do not directly impact the DNA sequence, and they reportedly might play a more important role in cancer onset than direct genomic mutations [1]. Furthermore, their maintenance is paramount to cancer survival and growth [2].

Several factors can modulate the epigenetic profile, both in physiological and transformed conditions. For example, it can be affected by environmental factors such as smoking, alcohol consumption, pollutants, and even circadian rhythm changes [3].

The concentration of certain metabolites, including ketone bodies [4], diallyl disulphide [5], folate acid and vitamin B12 [6], has also been recently reported to modulate epigenetic profiles. Additionally, a new body of work has been produced studying the possible epigenetic changes enacted by sphingolipids, which could further enrich the expanding role of these molecules in cell metabolism.

Sphingolipids are a class of seemingly structural fatty acids (FA) that have been recently found to also act as second messengers in many metabolic pathways and play an important role in cancer. In particular, ceramide and sphingosine 1-phosphate (S1P) possess anti-tumour and pro-tumour effects, respectively, with S1P exhibiting increased concentrations in many forms of cancer [7]. While ceramide promotes apoptosis and senescence, S1P upregulates proliferation, migration and cell survival, and thus constitutes a promising therapeutic target in cancer treatment [8].

In this review, we examine all current knowledge on the role of sphingolipids in epigenetic regulation, and lay the foundations for possible future studies. Furthermore, we draw attention to a new avenue of research, examining the effects of the accumulation of sphingolipids, induced by changes in the tumour microenvironment, on the epigenetic profile of cancer cells.

## 2. Epigenetic Modifications

Epigenetic changes are heritable genomic modifications that do not result in the alteration of the DNA sequence [9]. These can derive from several mechanisms, including the covalent modification of DNA (e.g., methylation) or histone proteins, and micro-RNA (miRNA)-mediated gene silencing. The proper regulation of epigenetic changes is fundamental in normal cell development.

DNA methylation is a common mechanism of epigenetic regulation, as methylation profiles are accurately inherited by daughter cells in physiological conditions [10]. Methylation typically occurs on cytosines located at genomic regions known as CpG islands, which are rich in CpG sites: stretches of DNA sequence where a cytosine is followed directly by a guanine. The reaction is catalysed by three enzymes called DNA methyltransferases. The specific effects of methylation vary depending on the targeted portion of each gene: methylated cytosines in the gene promoter generally result in downregulation through coiling the DNA around histones, rendering said DNA transcriptionally inactive [11]; on the other hand, the methylation of cytosines in the coding region may lead to an increased or decreased expression, depending on the case [11,12,13].

The modification of histone proteins is also one of the most studied mechanisms in epigenetics. DNA mostly exists in a compact structure, wrapped around nuclear proteins known as histones, which themselves group together to form a nucleosome. There are five different families of histones: H1/H5, H2A, H2B, H3 and H4, each containing several members. The nucleosome is formed of two of each: H2A, H2B, H3 and H4, organised in heterodimers, while H1 locks the DNA into place by binding at the entry and exit points in the nucleosome [14]. Several enzymes can modify histones by adding or removing functional groups, leading to changes in gene accessibility and, thus, transcription. These modifications generally occur at the N-tail of histones, and can include acetylation, methylation, phosphorylation, ADP-ribosylation, SUMOylation and ubiquitination [15,16].

Acetylation, in particular, is the most widely studied, and involves the removal of an acetyl group from acetyl-coenzyme A (acetyl-CoA) and its addition to the ε-NH+ terminal of lysine residues. This reaction is catalysed by histone acetyltransferases and results in an unravelling of chromatin around the targeted histone, increasing the transcription of the surrounding genes [17]. Vice versa, the deacetylation of histones occurs by histone deacetylases (HDACs), a class of enzymes that can deacetylate acetylated lysine residues in a histone amino acid sequence, inducing chromatin condensation, resulting in the downregulated expression of target genes [16]. HDACs can also deacetylate lysine residues in proteins other than histones, and have thus sometimes been called lysine deacetylases [18]. HDACs are divided into four families: Class 1 HDACs are ubiquitously expressed, predominantly nuclear and include HDACs 1, 2, 3 and 8 [19]; Class 2a HDACs can shuttle between nucleus and cytoplasm after phosphorylation, and include HDACs 4, 5, 7 and 9 [20]; Class 2b is comprised of HDAC 6, which is cytoplasmic [21], and HDAC 10, which can localise both to the nucleus and cytoplasm [20]; finally, HDAC11 is the only Class IV HDAC, and shows higher expression in the brain, heart, muscles, kidney and testes [22,23]. HDACs 1 and 2 are almost identical and can be found in several repression complexes such as sin3, nuRD, CoREST and PRC2 [24].

Histone methylation is another common type of modification, and also occurs at lysine residues. The reaction is catalysed by the enzyme histone methyltransferase, which can transfer a methyl group from S-adenosyl methionine to the target lysine. Like DNA methylation, the effects of histone methylation can vary between up- and downregulation, depending on the targeted histone and lysine [25]. Meanwhile, phosphorylation can instead occur at serine and tyrosine residues, and is important in the regulation of several important cellular processes, including cell cycle, genomic expression, and DNA repair. There is evidence of crosstalk between histone methylation and phosphorylation at certain sites [25]. ADP-ribosylation, sumoylation and ubiquitination are less known, overall. ADP-ribosylation is catalysed by ADP-ribosyltransferase and may be involved in the DNA damage response [26]. Sumoylation is the attachment of a protein, known as small ubiquitin-like modifier, to histone lysine residues, and generally results in suppressed transcription, though new functions have been recently explored [27]. Finally, ubiquitination is the addition of a ubiquitin molecule, once again on a lysine residue, and is mainly associated with the DNA damage response, though it also plays a role in regulating expression through the ubiquitination of H2A and H2B [28].

Lastly, another kind of epigenetic regulation is that induced by miRNAs. miRNAs are small non-coding RNA molecules that possess antisense codons specific for a number of target mRNAs. A miRNA duplex can recruit the RNA-Induced Silencing Complex through its sense codon, which is then degraded, and guide said complex to a target mRNA, promoting its degradation [29]. Conversely, some miRNAs can instead bind the 5’ UTR of a target mRNA to enhance its translation [30]. As such, miRNAs are able to modify protein levels without altering the normal genetic profile of the cell.

## 3. Epigenetics in Cancer

Methylation profiles can differ greatly between healthy and cancer cells, the latter usually exhibiting hypermethylation-mediated silencing of tumour-suppressor genes. Common targets of intragenic hypermethylation are *p16*, *MGMT*, *APC*, *MLH1* and *BRCA1* [31]. Hypermethylation can also lead to loss of imprinting. For example, the aberrant methylation of the *IGF2/H19* locus results in the overexpression of growth factor IGF2, which has been linked to the progression of colorectal and gastric cancer, as well as Wilms’s tumour and osteosarcoma [32,33,34]. However, despite the aforementioned hypermethylation of certain genes, total methylation is generally decreased in cancer cells [35], with most demethylation occurring in intragenic and intergenic regions rich in repetitive and transposable elements [36]. This can, for example, lead to the overexpression of the L1NE1 retrotransposon, a known oncogene, with increased DNA mutagenesis [37]. The maintenance of these altered methylation profiles is key for cancer development [2], and in fact, said profiles have been shown as a reliable way to diagnose specific types of bone sarcomas [38].

Like for the methylation profile, cancer cells present a different histone modification profile compared to healthy ones. For example, decreased monoacetylated and trimethylated forms of H4 compared to normal cells are observed in many human cancers [39].

Epigenetic modifications, including a different acetylation profile in histones, may be related to cancer aggressiveness. In osteosarcoma, for example, H3K27 acetylation activates the collagen type VI alpha 1 protein, promoting lung metastasis [40]. Histone modifications are also involved in the DNA damage response (DDR). DDR is an important factor in cancer development and progression, since it is often dysregulated [41]. For example, following a double strand break, a variant of H2A called H2AX is rapidly phosphorylated, which was shown to induce the accumulation of proteins linked to DNA repair, such as MDC1, RNF8, RNF168, 53BP1 and BRCA1 [42].

Finally, there have been countless studies on the roles of different miRNAs in cancer, both as pro-tumour and anti-tumour molecules affecting proliferation, survival, migration and angiogenesis [43]. For example, miR-17-92, a miRNA often overexpressed in several types of tumour, was found to promote the expression of E2F, a transcription factor heavily involved in inducing cell proliferation [44]. Another good example is the lack of balance in the several p53-regulated miRNAs, once again observed in many kinds of cancer [43]. Additionally, SNPs at the binding sites of certain miRNAs have been shown as viable biomarkers of gastric and colorectal cancer, highlighting the importance of miRNA regulation in maintaining normal cell homeostasis [45].

## 4. The Sphingolipid Pathway

**Figure 1:**
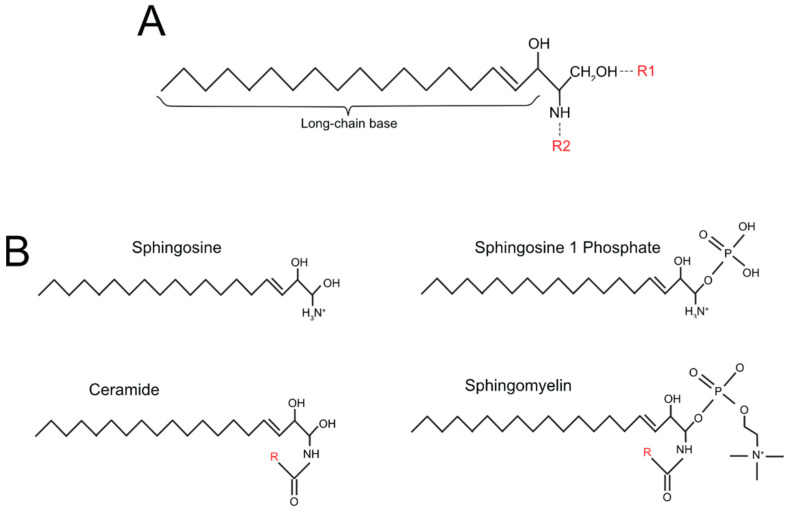
Basic structure of sphingolipids (**A**) and of the four more studied sphingolipids (**B**).

Sphingolipids are small molecules consisting of three main structural elements: a sphingoid long-chain base (lcb), usually sphingosine, sphinganine, or 4-hydroxysphinganine; an FA attached via amide bond to the C2 of the lcb; a head-group, which can be a sugar (glycosphingolipids) or phosphorylcholine, usually attached to a hydrophilic region. The basic structure of sphingolipids is shown in Figure 1A. The structure of the most studied sphingolipids is shown in Figure 1B, whereas additional information on the structure of more complex and less commonly studied sphingolipids is detailed in Table 1 [46].

**Figure 2:**
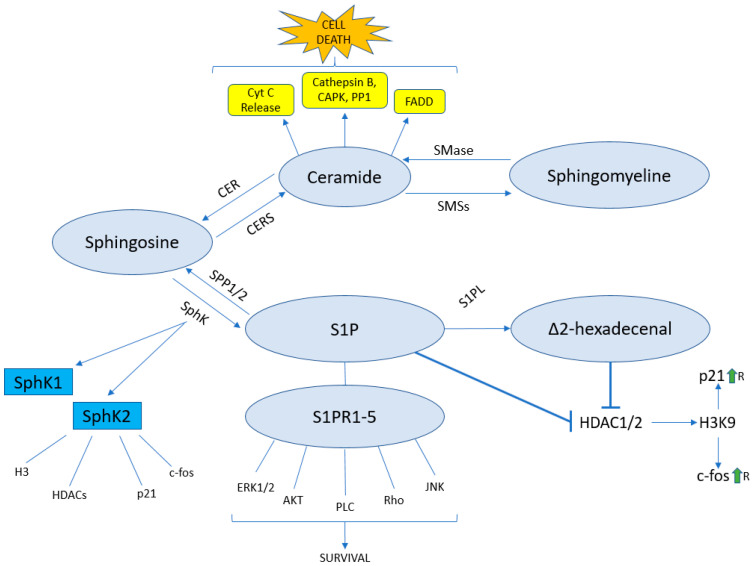
Schematic representation of the sphingolipid pathway. Sphingomyelin is converted by sphingomyelinase into ceramide, which then can induce cytochrome C release and increase expression of cathepsin B, CAPK, PP1 and FADD, resulting in cell death. Ceramidase converts ceramide into sphingosine, which can either be reconverted into ceramide via ceramide synthases, or be phosphorylated to S1P by sphingosine kinases. S1P mediates its cell survival effects by interacting with g-protein-coupled receptors S1PR1-5, resulting in activation of several pathways, and by inhibiting HDACs 1 and 2, increasing H3K9 acetylation and upregulating target genes. S1P is cleaved by S1P lyase into Δ2-hexadecenal, which might also be capable of inhibiting HDACs 1 and 2.

Ceramide occupies a central spot in the sphingolipid pathway (Figure 2) and is also the first signalling sphingolipid. It presents no head-group and can be generated de novo, starting from acyl-CoA. Different acyl-CoAs can be used, resulting in ceramides of various length [47]. The addition of a phosphate group to the sphingosine or ceramide yields S1P and ceramide-1-phosphate, respectively (Table 1). Furthermore, complex sphingolipids can be synthesised through ceramide modifications. The addition of a phosphocholine group to ceramide yields sphingomyelin, but the addition of glucose or galactose to ceramide yields glycosphingolipids and sulfatides (Table 1)

Through its pro-apoptotic pathways, ceramide can induce cytochrome C release and increase the expression of proteins such as cathepsin B, cAMP-dependant protein Kinase, protein phosphatase 1 and FAS-associated death domain [48]. This implies an important role for ceramide in cancer suppression, and in fact, its levels are decreased in many forms of cancer [49,50]. Interestingly, ceramide 1-phosphate, which is generated through the phosphorylation of ceramide by ceramide kinase, has pro-survival and pro-growth effects instead [51]. Ceramide can be converted into sphingosine by ceramidase, or into sphingomyelin by sphingomyelin synthases [52,53].

Sphingomyelin is a structural sphingolipid especially present in membrane microdomains, such as lipid rafts. Following cellular stimuli enacted by 1-α,25-dihydroxivitamin D3 and TNF-α, sphingomyelin is hydrolysed by sphingomyelinase to form ceramide and phosphocholine [54].

Sphingosine can be converted into ceramide by a ceramide synthase, or phosphorylated to S1P by a sphingosine kinase (SphK). There are two sphingosine kinases (SphK1 and SphK2), localised respectively in the cytoplasm and nucleus [55]. SphKs are activated after phosphorylation by extracellular signal-regulated kinases 1 and 2 (ERK1/2) in the cytoplasm or protein kinase D in the nucleus [56,57]. Additionally, SphK2 is often overexpressed in many types of cancer [58,59,60].

S1P is an important signalling sphingolipid that can mediate its pro-survival effects by interacting with five g-protein-coupled receptors, called S1P receptors 1-5 (S1PR1-5) [61], leading to the activation of signalling pathways such as ERK1/2 [62]; AKT [63]; PLC [64] and Rho [65]. Higher S1P levels have been detected in many types of cancer, and are possibly correlated with SphK2 overexpression [66]. S1P can be reconverted into sphingosine by S1P phosphohydrolases 1 and 2, or be irreversibly cleaved into Δ2-hexadecenal (Δ2-HDE) and ethanolamine phosphate by the enzyme sphingosine 1-phosphate lyase (S1PL) [67,68].

While Δ2-HDE was long considered just a by-product of S1P catabolism, very recent studies have linked it to different signalling pathways, such as the JNK-dependant induction of apoptosis and growth inhibition [69,70].

## 5. The Sphingolipid Pathway and Epigenetics

### 5.1. Sphingolipids and HDAC

**Figure 3:**
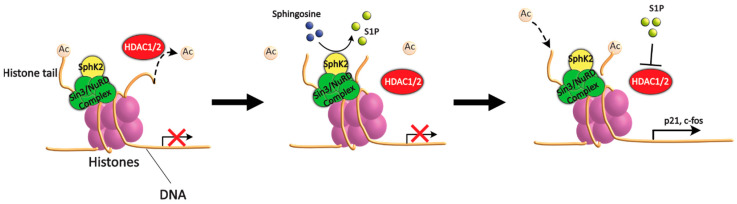
Schematic representation of HDAC inhibition by S1P. HDAC1/2 deacetylate histones as part of the Sin3 or NuRD repressor complexes localised at the promoters of target genes. SphK2 binds to said repressor complexes. SphK2 then synthetises S1P that, in turn, binds to and inhibits HDAC1/2, preventing deacetylation and thus increasing overall gene expression.

Both S1P and SphK2 have been demonstrated to be epigenetic regulators. In particular, a recent study has shown that, in the nucleus, S1P may function as an HDAC1 and HDAC2 inhibitor, leading to histone 3 (H3) acetylation [71]. Specifically, the authors found that SphK2 can associate to H3 and/or HDAC1/2, and the S1P it synthetises can bind to and inhibit HDAC1/2, preventing histone deacetylation (Figure 3). 

This results in an increased acetylation level, in particular of histone 3 (H3) lysine 9 (K9), but also H4-K5 and H2B-K12, leading to the activation and expression of several genes [71]. Intriguingly, SphK2 was shown to localise preferentially at said genes. Among these, we find the genes codifying for the cyclin-dependant kinase inhibitor p21, which is known to regulate cell-cycle progression, DNA replication and apoptosis [72], and the transcriptional regulator c-fos, a proto-oncogene associated with cell proliferation, differentiation and survival, as well as hypoxia response, angiogenesis and metastasis [73]. Additionally, in another study, the inhibition of HDAC1/2 by S1P has been linked to Ca^2+^ homeostasis. Both studies also reported that the silencing of SphK2 or overexpression of S1PL abrogated the inhibition of HDAC1/2 and resulting transcriptional regulation [71,74].

Therefore, according to the studies above, S1P and SphK2 are both active epigenetic regulators that can induce the inhibition of HDAC1/2, resulting in increased histone acetylation and the expression of certain genes.

### 5.2. Δ2-HDE as a Novel Epigenetic Regulator

S1PL can catabolise S1P into different by-products, including Δ2-HDE, that in turn have different biological activities. A study from 2005 reports that both SphK1 and S1PL are necessary to induce the increased proliferation normally associated with S1P, suggesting that a by-product of S1P catabolism might be at least partially responsible for this effect [75]. Recently, Δ2-HDE was shown to possess a number of regulatory functions. Kumar et al. demonstrated that the abundance of Δ2-HDE in HeLa cells causes detachment, cytoskeletal reorganisation and apoptosis, through the activation of MLK3 and JNK [69]. Amaegberi et al. later reported that Δ2-HDE can induce the activation of the p38 and ERK1/2 pathways, in addition to JNK, causing a decrease in proliferation in a dose-dependent manner. Furthermore, said decrease was shown to not be correlated with lipotoxicity from an excess of Δ2-HDE [70].

Given these novel discoveries, and the recent attention to epigenetic changes enacted by S1P, investigating Δ2-HDE for those same effects was the logical next step. A very recent study has shown a promising connection between Δ2-HDE and HDACs [76]. It was reported that, in lung cells infected with *Pseudomonas aeruginosa*, Δ2-HDE was capable of inhibiting HDAC1/2 activity, even in the absence of S1P. The effect was dose-dependent, with increased H3K9 and H4K5 acetylation, although interestingly, excessive doses of Δ2-HDE (>1 µM) led to decreased acetylation instead. Furthermore, all these effects were abrogated in cells lacking S1PL, even in the presence of S1P. Δ2-HDE was also shown to be able to form Lys adducts with HDAC1 in vitro. These results seemingly contrast with a previous article, which claimed that S1PL overexpression abrogated S1P-mediated HDAC1/2 inhibition [74]. Although the study by Ebenezer et al. did not use a cancer model, *Pseudomonas aeruginosa* infection has been previously reported to upregulate nuclear S1P [77]. Given the well-documented increase in S1P concentration in many types of cancer, investigating Δ2-HDE epigenetic activity in tumours could prove worthwhile.

In conclusion, Δ2-HDE, a compound generated by the S1PL-mediated lysis of S1P, was recently shown to possess a number of biological functions, and may possibly play a role in epigenetic regulation through the inhibition of HDAC1/2.

### 5.3. Ceramide and Epigenetics

Ceramide has opposite effects to S1P, activating pro-apoptotic pathways. This pattern seemingly holds true in regard to ceramide-mediated epigenetic changes, which play a role in counteracting tumour progression.

A recent study has shown that ceramide can inhibit the inhibitor 2 of protein phosphatase 2A (I2PP2A), resulting in increased H4 acetylation and decreased tumour progression [78]. I2PP2A is an inhibitor of the serine/threonine phosphatase 2A overexpressed in many forms of cancer, and is associated with cancer formation and progression [78]. Additionally, it is also a known inhibitor of acetyltransferases, negatively regulating H4 acetylation [79].

Another article reported that ceramide upregulates ciliogenesis in normal cells, specifically Madin–Darby canine kidney cells, possibly through promoting tubulin deacetylation, since trychostatin A, an HDAC inhibitor, exhibited similar effects [80]. Since cilia expression is abnormal in many types of cancer, this ceramide-mediated secondary epigenetic regulation shows yet another tumour-suppressant effect associated with the sphingolipid [81,82].

Finally, a study on adenocarcinoma in lung cells has shown that ceramide can bind to HDAC1 and promote the deacetylation of Sp3, a transcription factor heavily involved in human telomerase reverse transcriptase (hTERT) regulation, resulting in decreased expression of the latter. It should be noted that only C18, synthesised by CerS1, could downregulate hTERT by binding to HDAC1, and not C16 produced by CerS5 and CerS6, suggesting different roles for ceramide isoforms based on their carbon chain length [83]. This once again shows how ceramide and S1P can enact opposite effects, with the former promoting HDAC-mediated deacetylation and the latter decreasing it.

Thus, a number of studies have associated ceramide with different mechanisms of epigenetic regulations, often with opposite effects to S1P, including reduction in tumour progression and proliferation, and even the activation of HDAC1.

## 6. The Tumour Microenvironment and Epigenetic Balance

Recently, cancer research has shifted from looking at tumours as an isolated phenomenon to considering their interactions with surrounding cells and molecules. These are influenced by cancer cells into forming an area called tumour microenvironment (TME). The TME is altered by cancer cells to support growth, proliferation and protection from apoptosis [84]. The TME can itself be divided in stromal components, such as immune cells, mesenchymal stem cells (MSC), fibroblasts and epithelial cells and chemical components, which includes phenomena such as hypoxia and extracellular acidosis. In addition to being important for cancer development and resistance to treatments [85,86], the chemical TME has also been shown to both be a target and an inducer of epigenetic changes, in part through the accumulation of lipids, including sphingolipids, inside the cell, and is thus of particular interest to this review [85,86,87,88].

### 6.1. Hypoxia and the Sphingolipid Pathway

Hypoxia is a common phenomenon in solid tumours, which ensues when existing capillaries cannot grant a sufficient oxygen supply. This usually occurs when the tumour’s diameter exceeds 1 mm, but it is possible for a tumour to possess areas of intermittent hypoxia [89]. Low extracellular oxygen concentration causes a metabolic switch from oxidative phosphorylation (OXPHOS) to glycolysis through the activation or inhibition of the expression of specific genes associated with glycolytic metabolic pathways, such as the glucose transporters [85]. The regulation of the expression of these genes is determined by hypoxia-inducible factor (HIF), a transcription factor that can regulate the response to hypoxia. HIF is composed of two subunits, HIF-α and HIF-β [90]. HIF-α consists of a basic helix-loop-helix domain, two different Per, Ahr/ARNT, Sim (PASA; PASB) domains that are thought to be involved in HIF-αβ heterodimerisation and a c-terminal transactivation domain [91]. HIF-α can exist in two different isoforms, called 1α and 2α, which are respectively ubiquitously expressed, and only present in highly vascularised organs rich in hypoxic tissues. HIF-α normally exists in a hydroxylated form, making it an ubiquitination target, and thus leading to rapid degradation [92]. The enzyme responsible for HIF-α hydroxylation, HIF prolyl-hydroxylase, requires oxygen as a cofactor, and is thus inhibited in hypoxic conditions [93]. HIF-α is then free to migrate to the nucleus, where it heterodimerises with HIF-β [92]. The dimer can then modulate transcriptional activity, usually through binding sections known as hypoxia response elements on its target genes, which include *EPO*, *VEGF*, *HO-1*, *ADM* and *Glut-1* [94].

Hypoxia has generally been shown to lead to a worse prognosis in cancer [95,96] since it can lead to several changes in cancer cells, including altered gene expression, lowered apoptosis, epithelial–mesenchymal transition, metastasis and drug resistance. Interestingly, once the switch has happened, tumours retain a preference for glycolysis even in aerobic conditions, a phenomenon known as the Warburg effect [97]. A number of these effects are likely to be caused by changes in the epigenetic profile. For example, hypoxic conditions can lead to increased H3K4 and H3K36 trimethylation, resulting in chromatin rearrangement [98]. However, hypoxia can also activate several jumonji-type histone lysine demethylases, promoting the demethylation and thus altering the expression of several genes [99]. Additionally, hypoxia can induce the methylation of the chromatin remodelling protein Pontin, which is often overexpressed in cancer, by stabilising G9a, a methyltransferase, resulting in the hyperactivation of several HIF-α target genes [100]. Furthermore, HIF-1α can promote the recruitment of the histone lysine acetylase TIP60 to chromatin, leading to the acetylation of HIF-1α target genes in colorectal cancer [101].

Hypoxia has also been shown to alter lipid metabolism, including sphingolipids. A very recent study on pancreatic ductal adenocarcinoma has shown how genes related to lipid metabolism are abnormally expressed in cancer cells under hypoxic conditions, with different expression clusters leading to changes in survival time. Of particular interest, cluster 1 showed high levels of both SphK1 and SphK2 [102]. In the same year, another group confronted lipidomics from HCT 116 colon carcinoma cells grown in a 2D monolayer and a 3D spheroid model. Spheroids are used to simulate phenomena that occur in solid tumours, but cannot be replicated in 2D cultures, such as hypoxia. Interestingly, the results of the spheroids’ lipidomic show an increased production of sphingolipids, compared to that of the monolayer culture [103]. Conversely, sphingolipids have been shown to interact with the cellular response to hypoxia. In a recent study, immortalised renal interstitial fibroblasts stimulated with S1P were shown to enhance the stabilisation of HIF-2α, with a consequent increase in erythropoietin expression [104]. Judging from the cited body of work, it is logical to think that hypoxia and sphingolipids can interact with each other in a variety of ways, and it is worthwhile to examine their crosstalk from the point of view of epigenetics.

**Figure 4:**
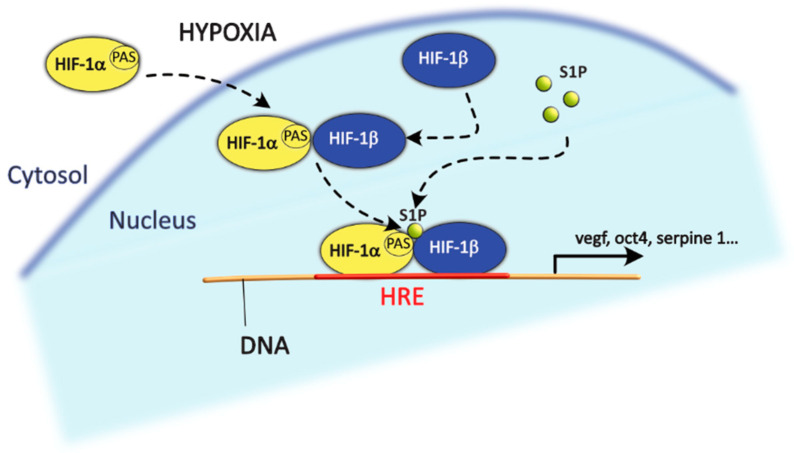
Schematic representation of the effect of S1P on HIF-1αβ. S1P can bind to the PAS domain of HIF-1α after the latter forms a heterodimer with HIF-1β, resulting in stabilisation of the heterodimer and increased transcription of HIF-1αβ target genes.

In a quite recent study on breast cancer, SphK2 was shown to associate with HIF-1α in repression complexes and to localise at the promoters of HIF target genes. The S1P-mediated inhibition of HDACs can lead to an increased overall acetylation of genes codifying for HIF-1α, promoting its expression. Furthermore, it can also cause the acetylation of the VEGF gene, an important target of HIF-1α, making it available for transcription and possibly allowing HIF-1α to bind to its hypoxia response element. Furthermore, S1P can bind directly to the PAS region of HIFα, resulting in higher protein stability and, thus, increased transcription of HIF target genes such as *VEGF*, *SERPINE1*, *OCT4*, *ALKBH5* and *CD44* in hypoxic conditions (Figure 4). Importantly, the silencing of SphK2 abrogated these effects in both normoxic and hypoxic conditions [105].

In conclusion, hypoxia can induce a large number of epigenetic changes, and thus S1P and SphK2, by upregulating HIF-1α expression and stability, can be considered indirect mediators of the described changes.

### 6.2. Acidosis and the Sphingolipid Pathway

**Figure 5:**
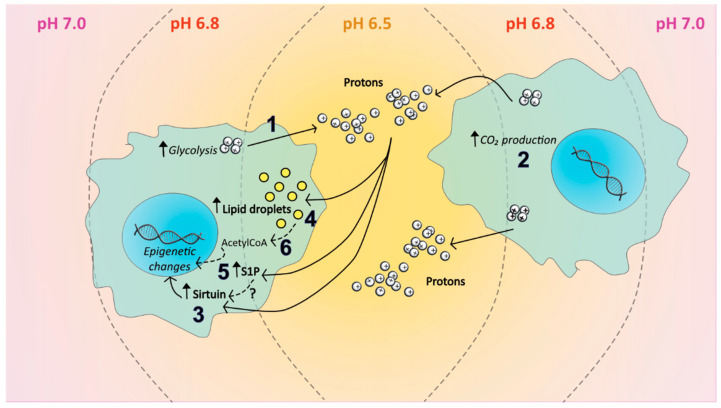
Possible mechanisms induced by acidosis in the TME. Extracellular acidosis results from the extrusion of protons in the TME from highly glycolytic cancer cells (1), or by the production of CO_2_ from adjacent cells (2). Acidosis, in turn, stimulates sirtuin(s) activity (3) and induces stress in tumour cells, which causes the accumulation of lipids inside the cell, in the form of lipid droplets, (4), and an increase in intracellular S1P (5) and Acetyl-CoA (6). The increased Acetyl-CoA and S1P concentration may lead to inhibition of HDAC1/2 and overexpression of SIRT1, thereby resulting in epigenetic changes.

As a consequence of hypoxia, or due to high glycolytic activity caused by the Warburg effect, high concentrations of protons accumulate in the cytosol and are readily transported passively or actively out of the cancer cell, causing extracellular acidosis (pHe) and the formation of an acidic TME [106] (Figure 5(1)). Tumour acidosis is a dysregulation of pH balance, wherein the normal gradient between intracellular and extracellular pH (pHi and pHe) is inverted, with pHe becoming acidic (6.5–7) and pHi alkaline (>7.4). In more detail, to ensure NAD+ regeneration in anaerobic conditions, the enzyme lactate dehydrogenase A converts pyruvate into lactate, which is then pumped outside the cell by different mechanisms. Among these, the monocarboxylate transporter 4, a lactate/H+ symporter, causes the accumulation of H^+^ ions outside the cell, thus decreasing the pH. Additionally, the increased consumption of ATP necessary to satisfy tumour metabolism can also lead to the excessive production of H^+^ ions. Thirdly, pHi is kept at an alkaline level by Na^+^/HCO^3−^ co-transporters, which provide bicarbonate to titrate-excess intracellular H^+^. Finally, cancer cells in the TME, while not exhibiting a metabolic switch, can still participate in decreasing pHe through the production of CO_2_ during OXPHOS [87,107,108] (Figure 5(2)). Interestingly, acidosis has been shown to induce genetic remodelling, including epigenetic changes, since it can increase the NAD+-dependent activity of histone deacetylases sirtuin 1 and 6 [87] (Figure 5(3)), which in turn can upregulate HIF-2α expression and downregulate HIF-1α and acetyl-CoA carboxylase [109,110]. In a collaborative study with Chano et al., we also investigated the acid-induced epigenetic changes in osteosarcoma cells. In this work, we tested whether or not there was a change in HDAC1/2 activity and/or H3K9 acetylation in osteosarcoma cells, compared to normal MSC. Interestingly, while controls presented an increased acetylation under acidosis, osteosarcoma cells did not show any significant change. This led us to believe that osteosarcoma cells have adapted to maintain a higher epigenetic stability compared to MSC [88]. This adaptation may possibly derive from an increased availability of metabolites that can serve as substrate for histone acetylation. It is a matter of fact that acidosis is accompanied by abnormal lipid metabolism in cancer, accumulations of lipids inside the cell, and compartmentalisation of accumulated lipids in droplets (Figure 5(4)) to prevent free FA-mediated cytotoxicity [87,111,112]. The accumulated lipids can then be used to form membranes for intracellular signalling, such as for ceramide and S1P (Figure 5(5)), or be oxidised to acetyl-CoA (Figure 5(6)) to generate energy and allow non-enzymatic acetylation [113]. Notably, in HUVEC cells, S1P upregulates Sirtuin 1 that, in turn, stimulates angiogenesis, although the exact mechanism, and whether or not it also occurs in cancer cells, is unknown [114]. It is likely that, in these cells as well as in cancer cells, the increased S1P accumulation induced by acidosis can result in higher Sirt1 expression on the one hand and the inhibition of HDAC1/2 on the other, although this has never been demonstrated.

However, although the data obtained so far are quite suggestive and promising, very few studies have been conducted in this area. Particularly unexplored is the role of Δ2-HDE in acidic conditions as well as the impact on the epigenetic profile of acid-induced lipid accumulation, as the latter leads to an increase in intracellular FA-derived acetyl-CoA that could be employed for histone acetylation, in addition to the already documented mitochondrial non-enzymatic hyperacetylation in tumours [113].

In conclusion, extracellular acidification is an important phenomenon in tumours, and can lead to several epigenetic changes. In addition, it can promote the intracellular accumulation of lipids, including S1P, which could possibly further alter the epigenetic profile due to S1P-mediated HDAC1/2 inhibition, even though this mechanism has yet to be studied.

## 7. Conclusions

Recent studies on several types of disease, including cancer, have revealed a number of novel roles of sphingolipids in cell metabolism. The regulation of epigenetic balance is the latest frontier in understanding the full effect of these molecules, with a particular focus on their interactions with histone deacetylases. However, this topic of research is as of yet in its infancy, and many avenues of study remain relatively unchallenged.

Tumour acidosis has been shown to play a crucial role in cancer progression and resistance to treatment. With hypoxia being a major cause of acidosis, and the recent evidence of the interaction between SphK2, S1P and HIFα, focusing more closely on how sphingolipid-mediated epigenetic regulation shifts in an acidic TME, is an interesting possibility. Additionally, according to the most recent data, Δ2-HDE, a novel player in sphingolipid metabolism, has been reported to interact with HDACs, but said interaction remains unexplored in cancer and thus presents several avenues for research, both in normal and acidic conditions. These further studies may serve to elucidate its role in the regulation of epigenetic balance.

All in all, the effects of sphingolipids on epigenetic changes are a novel topic that presents many enticing opportunities, both to thoroughly explore the subject and to later connect it to possible new treatments. To our knowledge, no review on the topic has been published previously, and thus this work may be helpful to researchers in planning future studies on sphingolipids and epigenetic changes in cancer.

## Figures and Tables

**Table 1:** Structures of sphingolipids.

Simple Sphingolipids	R1 Group (See Figure 1A)	R2 Group (See Figure 1A)
*sphingosine*	OH	H
*sphingosine-1 phosphate* (S1P)	PO_4_	H
*ceramide*	OH	O + Fatty acid residue
*ceramide-1-phosphate* (C1P)	PO_4_	O + Fatty acid residue
**Complex sphingolipids**		
*Sphingomyelin*	phosphocholine group	O + Fatty acid residue
*Cerebroside*	single sugar residue	O + Fatty acid residue
*Globoside*	di, tri, tetra-saccharide residue	O + Fatty acid residue
*Sulfatide*	single sugar residue + sulphate group	O + Fatty acid residue
*Ganglioside*	oligosaccharide residue + sialic acid	O + Fatty acid residue

## Data Availability

No new data were created or analysed in this study. Data sharing is not applicable to this article.

## Abbreviations

Fatty acid (FA); sphingosine 1-phosphate (S1P); micro-RNA (miRNA); acetyl-coenzyme A (acetyl-CoA); histone deacetylase 1 (HDAC1); histone deacetylase 2 (HDAC2); DNA damage response (DDR);); long-chain base (lcb); sphingosine kinase 1 and 2 (SphK1, SphK2); extracellular-signal-regulated kinase 1 and 2 (ERK1 and ERK2); called S1P receptors 1-5 (S1PR 1-5); Δ2-hexadecenal (Δ2-HDE; sphingosine 1-phosphate lyase (S1PL); histone 3 (H3); lysine 9 (K9); inhibitor 2 of protein phosphatase 2A (I2PP2A); human telomerase reverse transcriptase (hTERT); tumour microenvironment (TME); mesenchymal stem cell (MSC); oxidative phosphorylation (OXPHOS); hypoxia-induced factor (HIF); Per, Ahr/ARNT, Sim A and B (PASA, PASB); extracellular pH (pHe); intracellular pH (pHi).
